# Bacterial transmission within social groups shapes the underexplored gut microbiome in the lemur *Indri indri*

**DOI:** 10.1093/ismejo/wraf136

**Published:** 2025-07-25

**Authors:** Francisca Labisa-Morais, Daria Valente, Aitor Blanco-Míguez, Paolo Manghi, Albert Garcia-Valiente, Harilala Andriamaniraka, Federica Armanini, Francesco Asnicar, Chiara De Gregorio, Davide Golzato, Serena Manara, Monica Modesto, Elisa Piperni, Michal Punčochář, Donatella Scarafile, Valeria Torti, Luigimaria Borruso, Paola Mattarelli, Cristina Giacoma, Camillo Sandri, Caterina Spiezio, Nicola Segata, Mireia Valles-Colomer

**Affiliations:** Medicine and Life Sciences (MELIS) Department, Universitat Pompeu Fabra, Doctor Aiguader 88, Barcelona, Bcn 08003, Spain; Department of Life Sciences and Systems Biology, University of Torino, Via Accademia Albertina 13, Turin, To 10123, Italy; Department of Cellular, Computational and Integrative Biology (CIBIO), University of Trento, Via Sommarive 9, Trento, Tn 38123, Italy; Department of Cellular, Computational and Integrative Biology (CIBIO), University of Trento, Via Sommarive 9, Trento, Tn 38123, Italy; Research and Innovation Center, Fondazione Edmund Mach, Via Edmund Mach 1, San Michele all’Adige, Tn 38098, Italy; Medicine and Life Sciences (MELIS) Department, Universitat Pompeu Fabra, Doctor Aiguader 88, Barcelona, Bcn 08003, Spain; Tropical Agriculture and Sustainable Development Department, University of Antananarivo, BP 566, Antananarivo, 101, Madagascar; Department of Cellular, Computational and Integrative Biology (CIBIO), University of Trento, Via Sommarive 9, Trento, Tn 38123, Italy; Department of Cellular, Computational and Integrative Biology (CIBIO), University of Trento, Via Sommarive 9, Trento, Tn 38123, Italy; Department of Psychology, University of Warwick, Coventry, CV4 7AL, United Kingdom; Department of Cellular, Computational and Integrative Biology (CIBIO), University of Trento, Via Sommarive 9, Trento, Tn 38123, Italy; Department of Cellular, Computational and Integrative Biology (CIBIO), University of Trento, Via Sommarive 9, Trento, Tn 38123, Italy; Department of Agricultural and Food Sciences, University of Bologna, Viale G. Fanin 44, Bologna, BO 40127, Italy; Department of Cellular, Computational and Integrative Biology (CIBIO), University of Trento, Via Sommarive 9, Trento, Tn 38123, Italy; Department of Experimental Oncology, IEO European Institute of Oncology IRCCS (Istituto di Ricovero e Cura a Carattere Scientifico), Via Giuseppe Ripamonti 435, Milan, Mi 20141, Italy; Department of Cellular, Computational and Integrative Biology (CIBIO), University of Trento, Via Sommarive 9, Trento, Tn 38123, Italy; Department of Agricultural and Food Sciences, University of Bologna, Viale G. Fanin 44, Bologna, BO 40127, Italy; Department of Life Sciences and Systems Biology, University of Torino, Via Accademia Albertina 13, Turin, To 10123, Italy; Faculty of Agricultural, Environmental and Food Sciences, Free University of Bozen-Bolzano, Piazza Università 5, Bolzano, I-39100, Italy; Department of Agricultural and Food Sciences, University of Bologna, Viale G. Fanin 44, Bologna, BO 40127, Italy; Department of Life Sciences and Systems Biology, University of Torino, Via Accademia Albertina 13, Turin, To 10123, Italy; Department of Animal Health Care and Management, Parco Natura Viva, Garda Zoological Park, Località Figara 40, Bussolengo, Verona, VR 37012, Italy; Department of Animal Health Care and Management, Parco Natura Viva, Garda Zoological Park, Località Figara 40, Bussolengo, Verona, VR 37012, Italy; Department of Cellular, Computational and Integrative Biology (CIBIO), University of Trento, Via Sommarive 9, Trento, Tn 38123, Italy; Department of Experimental Oncology, IEO European Institute of Oncology IRCCS (Istituto di Ricovero e Cura a Carattere Scientifico), Via Giuseppe Ripamonti 435, Milan, Mi 20141, Italy; Medicine and Life Sciences (MELIS) Department, Universitat Pompeu Fabra, Doctor Aiguader 88, Barcelona, Bcn 08003, Spain; Department of Cellular, Computational and Integrative Biology (CIBIO), University of Trento, Via Sommarive 9, Trento, Tn 38123, Italy

**Keywords:** *I. indri* microbiome, metagenomics, microbial diversity, social transmission, soil microbiome

## Abstract

The *Indri indri* is a critically endangered lemur species that has not successfully been maintained or bred under human care. Investigating this lemur’s virtually unexplored gut microbiome will deepen our understanding of the species’ health determinants and inform conservation efforts. Through metagenomic assembly and integration into an updated reference database, we found the *I. indri* fecal microbiome remains largely uncultivated (cultivated species representing <0.1% relative abundance) and is largely specific to this primate species. After reconstructing 342 metagenome-assembled genomes encompassing 48 candidate species from a total of 22 samples (18 of which newly sequenced), we substantially improved microbiome mappability to 85% on average and found evidence for a proportionally large core microbiome. Social group membership emerged as the main determinant of both their taxonomic and functional gut microbiome composition. Using strain-level profiling, we detected extensive microbiome transmission within social groups, suggesting physical interaction is key in promoting microbiome acquisition. Strain sharing rates were highest between mothers and their offspring. Intergroup strain sharing was minimal and inversely correlated with geographical distance, aligning with the rare intergroup interactions and stable territory occupancy coupled with ongoing habitat fragmentation. No evidence of microbiome acquisition through geophagy was detected. These findings underscore the profound influence of social structure on microbiome transmission and composition in *I. indri*, and highlight the importance of considering social dynamics into research and conservation strategies.

## Introduction

More than half of non-human primate (NHP) species are classified as either vulnerable, endangered, or critically endangered by the IUCN red list [1], with their populations declining at alarming rates [2]. Efforts to characterize NHP gut microbiomes have revealed a high degree of host species specificity, and >90% of their microbial species remain uncultivated [3, 4]. Captivity is often associated with reduced gut microbiome diversity, only partially due to dietary changes [5, 6]. As microbiomes are crucial to host health, there is a growing consensus that they should be incorporated into conservation strategies [7, 8]. In addition to benefiting host fitness, wild animal microbiomes also offer potential for discovery of biotherapeutics [9] and, as our closest evolutionary relatives, NHP microbiomes may offer insights into human-microbe coevolution [10].

The *Indri indri* is an arboreal lemur endemic to Madagascar’s eastern rainforests that is critically endangered due to habitat loss from human activity [11]. Attempts to maintain or breed this lemur in captivity have been unsuccessful, suggesting unfulfilled ecological or behavioral needs under human care [12, 13]. They live in stable social groups of two to five individuals, composed of the adult reproductive pair and offspring [14, 15]. They are monogamous (forming stable, long-term bonds) and social groups display female dominance. Social groups occupy small and exclusive territories, with intergroup encounters being rare and usually resolved by vocal confrontation [16, 17]. Indeed, the *I. indri* produces loud calls to advertise their presence and territory occupancy [18]. Their folivorous diet consists mainly of immature leaves, with seasonal variation adding fruits, seeds, flowers, and bark [19], and frequently engage in geophagy (soil eating). Multiple hypotheses have been proposed for this behavior, including lowering gastric pH, neutralizing plant toxins, supplementing nutrient-poor diets, or diversifying their microbiomes [20, 21].

So far, few studies have examined the *I. indri* gut microbiome composition, all using reference-based approaches that preclude the identification of novel microbial diversity. Gut microbiome composition was assessed in four lemur species [22], including 17 *I. indri* from 10 different social groups, using a combination of amplicon (*N* = 29) and metagenomic sequencing (*N* = 4). The authors identified an unassigned *Prevotellaceae* genus as dominant and reported *I. indri* microbiomes as enriched in functions associated with fiber metabolism, plant secondary compound degradation, and aromatic compound breakdown - consistent with their diet. Another study using 16S rRNA gene sequencing on 18 *I. indri* from five social groups [23], identified social group and sex as the main covariates of gut microbiome composition. However, the phylum-level bacterial composition they observed diverged from that reported in the first study [24], which could be attributed to both technical factors and population differences. A third study focused on the fungal fraction of the microbiome (fungal ITS sequencing) in *I. indri* fecal samples (*N* = 9) and geophagy-associated soil (*N* = 7), finding shared fungal operational taxonomic units (OTUs) between them that may reflect microbial acquisition through soil consumption [21].

Besides their understudied microbial diversity, little is known about the acquisition and transmission mechanisms of the *I. indri* gut microbiome. In humans, the gut microbiome is seeded at birth by maternal transmission, and then shaped by transmission from proximate individuals [25]. Given this lemur’s limited intergroup contact and stable territory occupancy, social microbiome transmission may play a major role in shaping their gut microbiome. Indeed, most studies in lemurs so far found evidence for socially structured microbiomes [26–28]. Similarly, research on chimpanzees has shown that sympatry leads to microbial convergence [29], and that seasonal increase in sociability further promotes microbiome similarity [30]. In baboons, social group membership emerged as the strongest predictor of microbiome taxonomic and functional composition, with socially structured bacterial taxa being predominantly anaerobic and non-spore-forming, suggesting that microbiome similarities in social groups are due to microbiome transmission through physical contact [31]. Therefore, although there are clear hints supporting social microbiome transmission as a key factor shaping NHP microbiomes, this has yet to be confirmed with approaches that reach the necessary taxonomic resolution to allow microbial strain tracking. Here, we used metagenomic assembly to uncover uncharacterized diversity in the *I. indri* gut microbiome, to then investigate transmission within and between social groups together with microbial acquisition through geophagy using strain-level profiling.

## Materials and methods

### Study dataset and data collection

*I. indri* fecal samples (*N* = 18) and soil samples (*N* = 14) were collected in the Maromizaha forest in Madagascar (latitude 18°57′S and 19°00′S, longitude 48°26′E and 48°31′E), a medium altitude evergreen rainforest including a core zone (1175 ha), a buffer zone (725 ha), and an ecotourism zone (250 ha) [13]. Each sampled individual had previously been identified using natural marks, that is individual-specific traits that can be identified from a distance, such as the color and pattern of their fur patches, and their size. In addition, their singing patterns were used to tell males and females apart. The individuals belonged to six different social groups (labeled 1MZ, 2MZ, 3MZ, 4MZ, 6MZ, and 8MZ), achieving an average coverage of 72% of sampled individuals per group (1MZ: 2 out of 3 individuals, 2MZ: 4/4, 3MZ: 3/4, 4MZ: 2/5, 6MZ: 2/4, 8MZ: 4/6). Samples were further classified according to sex (female, male, or not available (NA) when sex identification was not possible due to early age), age (specified for individuals aged 6 years or below, older individuals were described as >6 years old as precise age information was unavailable), and family role (mother and father for the female and male of a reproductive pair, respectively, and progeny). Subjects were further categorized according to their age into infants (below one year of age), yearlings (1 to 2 years of age), and adults (>2 years of age, including both non-reproductive individuals and reproductive pair members [32]) (Table S1).

Location data of the *I. indri* social groups were recorded using GPS (global positioning system) coordinates during the year of 2018. Direct field observations were conducted from early morning until noon to align with their diurnal activity pattern [16, 17]. The ranging pattern of the *I. indri* is characterized by progressive directional displacements [16], that is the groups move through successive stationary areas where they stop. In these stationary areas, group members engage in activities such as feeding, resting, or sleeping, and may move within or between adjacent trees while maintaining group cohesion. For a new stationary area to be considered, an *I. indri* group had to interrupt their previous activity, move to a new area at least 20 m away, and stay there for at least 5 minutes. For each stationary area, the location of the center of the group members was recorded using a hand-held GPS (Garmin MAP 76CSX) with an accuracy of 5 meters [16, 17]. Of the social groups, three (4MZ, 6MZ, and 8MZ) resided either fully or partially within the Maromizaha ecotourism zone, with potential contact with tourists.

**Figure 1:**
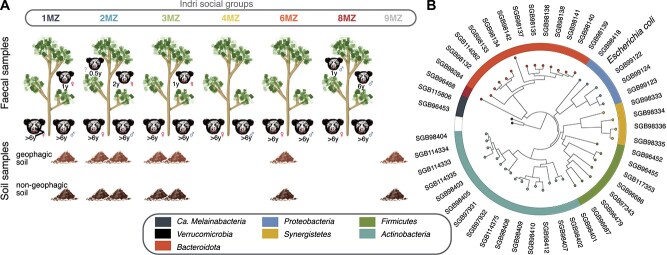
Uncovering the *I. indri* gut microbial tree of life. (A) Study design. Each social group is represented on a tree, noting their age and sex (when known), and with the reproductive pair depicted at the bottom. Soil samples are aligned with the social group in the area of collection. (B) The *I. indri* current gut microbial tree of life. For each candidate species with at least one reconstructed MAG, the MAG with the highest quality was selected as representative and used to build the phylogenetic tree based on a set of 400 universal marker genes using PhyloPhlAn 3 [47] (methods).

Soil samples were collected from geophagic sites, defined as where soil-eating events occurred (7 samples of groups 1MZ, 2MZ, 3MZ, 6MZ, 9MZ), and from non-geophagic sites nearby (7 samples of the same groups). The social group engaging in geophagic behaviors was recorded for each sampling site (Fig. 1A).

### Fecal and soil sample collection and storage

Sample collection was performed during a narrow time window (4 to 6 December 2018) to minimize potentially confounding seasonal variations, as previously described [23]. Approximately 2 g of feces were collected per sample immediately after defecation and only when a single animal was present to avoid misidentification, following the procedure previously described [21]. To minimize the risk of soil contamination, fecal material was collected from the center of fecal samples. The retrieved stool was transferred into DNA/RNA Shield Fecal Collection Tubes (Zymo Research R1101) with 9 ml of RNAlater (ThermoFisher Scientific) for sample stabilization and preservation. Collection tubes were sealed to prevent any cross-contamination [21] and subsequently stored in a laboratory refrigerator at 4°C until further analysis.

Geophagic sites were located at the base of fallen trees where the roots and surrounding soil are visible (uprooted trees), exposing lower soil horizons. Non-geophagic soil samples were collected from sites with similar characteristics to that of the corresponding geophagic site, located less than 20 m away from them. A superficial soil layer was removed prior to non-geophagic soil sampling to match the geophagic soil layer [23]. Soil samples were collected into 50 ml Falcon tubes, and subsequently stored in a laboratory freezer at −80°C until further analysis.

### Deoxyribonucleic acid extraction and metagenomic sequencing

Total DNA was extracted from fecal and soil samples using the DNeasy PowerSoil Kit (QIAGEN) according to the manufacturer’s procedures. DNA concentration was measured using the Infinite 200 PRO microplate reader (Tecan) and stored at −20°C. Sequencing libraries were prepared using the Illumina DNA Prep—(M) Tagmentation Kit according to the manufacturer’s guidelines, followed by a purification step with Agencourt AMPure XP beads (Beckman Coulter) at a 0.7× bead ratio. Sequencing was performed at the LaBSSAH–CIBIO Next Generation Sequencing Facility of the University of Trento, Italy, on a NovaSeq 6000 S4 v1.5 flow cell (Illumina), following manufacturer’s protocols.

### Metagenomic data pre-processing and quality control

Reads from stool and soil samples were pre-processed using the pipeline described in https://github.com/SegataLab/preprocessing. Shortly, metagenomic reads were quality controlled and reads of low quality (quality score < Q20), fragmented short reads (<75 bp), and reads with >2 ambiguous nucleotides were removed with Trim Galore (v0.6.6). Contaminant and host DNA were identified with Bowtie2 (v2.3.4.3) [33] using the—sensitive-local parameter, allowing confident removal of the Instrument’s spike-ins and host-associated reads (hg19 human genome release). Although only a scaffold is available for the *I. indri* genome (NIH BioProject accession PRJNA399458), its high identity to the human reference genome should allow removing host DNA. Only samples with more than 1 million reads after the preprocessing were included in the downstream analysis (*N* = 29, median = 46.9 million reads/sample, IQR = [29.3, 70.0] million reads/sample). Samples 1_1MZ, 10a_6MZ, and 12a_9MZ, all corresponding to geophagic soil sites, were thus disregarded. Read statistics of all samples (number of reads, number of bases, minimum, median, and maximum read length per sample are detailed in Table S1).

### Metagenomic assembly, creation of an *Indri indri* microbiome catalog, and a database for reference-based profiling

To assemble an *I. indri* metagenome-assembled genome (MAG) catalog, we used the 18 fecal samples newly sequenced in this study together with 4 samples previously sequenced [24]. In addition, we assembled publicly available fecal microbiome samples of other NHPs, as when integrated into a database for reference-based microbiome profiling, they might help profiling microorganisms that are not exclusive to this primate species. Therefore, beside including six metagenomic datasets [31, 34–38] already assembled in Manara *et al.* [4], we also assembled the metagenomic samples in Srivanathsan *et al.* (with 6 samples of *Presbytis femoralis*) [39]*,* Mc Kenney *et al.* (with 18 samples of *Varecia variegata*, *Lemur catta*, and *Propithecus coquereli*) [40], Greene *et al.* (including 8 samples of *Propithecus diadema* beside the 4 of *I. indri*) [24], Campbell *et al.* (including 160 samples of *Pan troglodytes*, *Gorilla gorilla*, and *Homo sapiens*) [41], Sharma *et al.* (with 23 samples of *G. gorilla*) [42], and Greene *et al.* (with 11 samples of *P. coquereli*) [22]. In addition, we added the MAGs released in Levin *et al.* (assembled from 51 samples from multiple species including 27 wild lemurs) [3].

Metagenomic assembly was performed with MEGAHIT [43] using default parameters. Assembled contigs longer than 1500 nucleotides were binned into MAGs using MetaBAT2 [44]. Quality control of all MAGs was performed with CheckM version 1.1.3 [45], and only medium- and high-quality MAGs [completeness ≥50% and contamination ≤5% for medium quality (MQ), completeness ≥90% and contamination ≤5% for high quality (HQ)] were retained to be included in the catalog used to create a custom reference database. 271 MAGs were retained from the newly sequenced dataset (256 of which from *I. indri* and the rest from soil), 974 MAGs from [39]*,* 666 from [40], 375 from [24], 716 from [41], 1013 from [42], and 106 from [22]. We followed the procedure described previously [46] to build a species-level genome bin (SGB) catalog expanding the vJan21 database. This way, we first applied the “phylophlan_metagenomic” subroutine of PhyloPhlAn 3 [47] on the MAGs to identify their closest SGB, GGB, FGB, and their MASH distances. MAGs were assigned to already existing SGBs, GGBs, and FGBs according to thresholds defined previously (5%, 15%, and 30% genetic distance, respectively) [48]. To the genomes not assigned to any existing SGB, a hierarchical clustering approach with average linkage was applied on the all-versus-all MASH distances using the “fastcluster” python package version 1.1.25. The resulting dendrogram was divided with cutoffs at 5%, 15%, and 30% genetic distance to define new SGBs, GGBs, and FGBs. SGBs containing at least one reference genome (kSGBs) were assigned the taxonomy of the reference genomes following a majority rule, up to the species level. SGBs with no reference genomes (uSGBs) were assigned the taxonomy of its corresponding GGB (up to the genus level) if this contained reference genomes, and of its corresponding FGB (up to the family level) if the latter contained reference genomes. If no reference genomes were present in the FGB, a phylum was assigned based on the majority rule applied on up to 100 closest reference genomes to the MAGs in the SGB as provided by “phylophlan_metagenomic”.

The MAGs in the catalog were also annotated following a procedure described previously [46]. Prokka (version 1.14) [49] was used to detect and annotate the coding sequences (CDS), and CDS were then assigned to a UniRef90 cluster [50] using a DIAMOND-based pipeline (available at https://github.com/biobakery/uniref_annotator). CDS that were not assigned to any UniRef90 cluster were clustered using MMseqs2 [51] following the UniClust90 criteria (“-c 0.80–min-seq-id 0.9” parameters) [52]. Based on the UniRef90 and UniClust90 annotations, a pangenome was generated for each SGB by collecting all the UniRef/UniClust90 clusters present in at least one of the genomes in the SGB. For each cluster, the representative sequence was randomly selected within all the genomes, and a coreness value was calculated based on the cluster prevalence within the 2 k highest quality genomes of the SGB. All uSGBs containing MAGs assembled in *I. indri* samples were kept, and uSGBs with MAGs assembled in samples of other host species were only retained if they had at least 1 HQ MAG or 3 MAGs. Marker genes for the SGBs newly assembled in the NHP datasets included into a custom MetaPhlAn 4 database following the procedure previously described [46].

### Inference of phenotypic and metabolic traits of the newly identified candidate species

Phenotypic traits and metabolic capabilities were predicted for all newly identified candidate species (SGBs) using Traitar (version 3.0.1) [53] on the 50% core genes (genes present in 50% of genomes available in the expanded SGB database) as in previous work [25]. Only annotations for which the *phypat* and the *phypat + PGL* classifiers (the second including additionally evolutionary information on phenotype gains and losses) annotations matched were kept.

The distribution of the metabolic pathway leading to production of the secondary bile acids deoxycholic acid (DCA) and lithocholic acid (LCA), the main bile acids produced by bacteria that display antimicrobial activity [54, 55] was assessed through the detection of the UniRef50 identifiers (Uniprot_042025 release) [50] annotated with the Enzymatic Commission numbers (EC numbers) in the seven-step pathway of bile acid 7α-dehydroxylation (MetaCyc pathway PWY-7754) [56]. Similarly, to detect antimicrobial peptides (AMPs) the APD3 database [57] was used to annotate the UniRef50 clusters in each of the genomes assembled.

### Phylogenetic tree of the *Indri indri*-associated microbial diversity

A phylogenetic tree of the MAGs assembled from *I. indri* metagenomic samples was built including the 256 MAGs reconstructed from the 18 individuals in the newly sequenced dataset plus 86 MAGs reconstructed from the four samples previously available [24]. The MAGs belonged to 48 different SGBs (41 of which were detected in the newly sequenced dataset). For each SGB, the MAG having the highest quality value defined as “completeness - 3 x contamination”, as established by CheckM [45], was selected as representative. The representative MAGs were used to build a tree using PhyloPhlAn 3 [47] by aligning the fasta files of their proteomes against the available set of 400 universal marker genes. Key-parameters used were: “--diversity high --fast --min_num_markers 50”. The tree was rooted on the longest internal branch and visualized with GraPhlAn [58].

### Species- and strain-level microbiome profiling

Species-level profiling was performed on all the samples with MetaPhlAn 4.0.6 [46] with default parameters and the custom database (available at http://cmprod1.cibio.unitn.it/biobakery4/metaphlan_databases/). Strain-level profiling was performed with StrainPhlAn 4.0.6 [46] using the same database, for all SGBs detected with MetaPhlAn or containing MAGs assembled in the samples in the dataset, using the following parameters: —phylophlan_mode fast—sample_with_n_markers (20 for SGBs with at least 50 markers and 10 otherwise) —secondary_sample_with_n_markers (20 for SGBs with at least 50 markers and 10 otherwise). Samples sequenced in the context of this study were considered as primary samples, whereas NHP samples in other studies were included as secondary samples to augment the dataset and obtain more reliable strain sharing events. Only resulting phylogenetic trees corresponding to SGBs that could be profiled at the strain level in at least 5 samples (2 of which in the target dataset) were kept. Out of a total of 97 SGBs, 37 were thus included in the strain sharing analysis. Normalized phylogenetic distances were calculated as leaf-to-leaf branch lengths normalized by the total branch length of the tree. As no NHP longitudinal samples were available, none of the SGBs were found in human longitudinal datasets [25], and no longitudinal shotgun metagenomic datasets with *I. indri* fecal samples are available, we used a species-specific conservative strain identity threshold corresponding to the 5th percentile of each species’ phylogenetic distance distribution. Strain sharing events were identified as pairs of strains with a pairwise phylogenetic distance below the strain identity threshold.

Interindividual strain sharing rates were calculated as the number of shared strains between two individuals divided by the number of shared SGBs profiled by StrainPhlAn in the two individuals as previously described [25]. To ensure robust estimates, only pairwise strain sharing rates between individuals sharing at least 5 SGBs were kept. Species transmissibility was calculated as the number of strain sharing events detected for a SGB divided by the total number of potential strain sharing events for that SGB based on the presence of a strain-level profile by StrainPhlAn. To ensure robust estimates, SGB transmissibility was only assessed for SGBs with at least 5 potential strain sharing events.

### Functional microbiome profiling

Functional profiling was performed with HUMAnN 3.8 [59], using default parameters and the Oct22 CHOCOPhlAnSGB database. MetaCyc [56] pathway definitions were reported as provided by HUMAnN.

### *Blastocystis* profiling

The presence of *Blastocystis* in metagenomic samples was assessed using a previously validated computational workflow [60] as previously described [61]. Nine reference genomes for eight different *Blastocystis* subtypes (ST1 [ST1_LXWW01], ST2 [ST2_JZRJ01], ST3 [ST3_JZRK01], ST4 [GCF_000743755 & ST4_BT1_JZRL01], ST6 [ST6_JZRM01], ST8 [ST8_JZRN01], and ST9 [ST9_JZRO01]) were mapped against metagenomic reads with Bowtie2 (v2.3). SAMtools (v1.19) and bedtools were then used to compute the breadth of coverage of each genome, and a sample is considered to be positive for a *Blastocystis* subtype if the respective genome has a breadth of coverage of at least 10%.

### Alpha diversity, beta diversity, and ordination

Species-level abundance matrices obtained in MetaPhlAn were centered log ratio-transformed using the codaSeq.clr function in the CoDaSeq R package (v0.99.6) [62], using the minimum proportional abundance detected for each species for the imputation of zeros. Only species with an average relative abundance of at least 0.001 were kept. Species-level richness (Observed) and diversity (Simpson’s) were determined using the estimate_richness function in phyloseq, and evenness (Pielou) was calculated as the Shannon index divided by the logarithm of the Observed richness [63]. A principal coordinate analysis (PCoA) on Aitchison distance (Euclidean distance between samples after centered log-ratio transformation) was produced with the ordinate and plot_ordination functions in phyloseq (v1.28.0) [64]. The association between metadata variables and distance matrices was assessed by PERMANOVA with the adonis function in vegan (v2.6.4) [65].

### Strain sharing networks

Unsupervised networks based on shared strains among individuals were visualized with the R packages ggraph (v2.2.1) [66] and tidygraph (v1.3.1) [67]. Centrality measures (degree centrality, eigenvector centrality, and betweenness centrality) were computed with the igraph (v2.0.2) [68] package. Degree centrality is defined as the number of connections held by a node, or individual in this case. Eigenvector centrality of a node illustrates the centrality of the nodes that are connected to it, meaning a node with high eigenvector centrality is connected to highly central nodes of the network. Betweenness centrality measures the fraction of shortest paths between any 2 other nodes that pass through that node.

### Spatial analysis

Spatial analysis and visual representation was performed with R packages sf (v1.0.16) [69], ggplot2 (v3.5.0) [70], and mapview (v2.11.2) [71]. The GPS waypoints were imported as decimal degrees (World Geodetic System 1984—WGS84) and projected to WGS 84 / UTM zone 39S [16]. Convex hulls, also known as minimum convex polygons (MCPs), were defined as the smallest convex geometry that encloses all coordinates in a social group. Territory size during the sampled period was estimated by computing the area of each group’s MCP, corresponding to the MCP100. The area of overlap between the MCPs of different groups was also computed, when applicable. The geometric centroid of each MCP was computed and used to calculate the distance between *I. indri* social groups.

### Statistical analysis

Statistical analyses and graphical representations were performed in R with packages vegan (v2.6.4) [65], phyloseq (v1.46.0) [64], ggplot2 (v3.5.0) [70], ggpubr (v0.6.0) [72], gplots (v3.1.3.1) [73]. Differences between two groups were assessed with Wilcoxon rank-sum tests. For more than two groups, the Kruskal–Wallis test with post-hoc Dunn tests was used. Correlations were assessed with Spearman’s tests. All tests were two-sided except where specified otherwise. Correction for multiple testing (Benjamini–Hochberg procedure, *P*_adj_) was applied when appropriate and significance was defined at *P*_adj_ < .05.

### Ethical compliance

The non-invasive methods used for fecal collections of wild indris adhere to the International Primatological Society (IPS) “Principles for the Ethical Treatment of Non-Human Primates”. Field data collection protocols were reviewed and approved by Madagascar’s Ministère de l’Environnement, de l’Écologie et des Forêts and Direction du Système des Aires Protégées (Research Permit N° 91/18/MEEF/SG/DGF/DSAP/SCB.Re). Field data collection protocols were also approved by GERP (Groupe d’Étude et de Recherche sur les Primates de Madagascar), the association governing research in the Maromizaha New Protected Area.

## Results and discussion

### Metagenomic dataset for assessing *Indri indri* gut microbiome diversity, acquisition, and transmission patterns

Fecal samples of 18 *I. indri* belonging to six different social groups were collected in the Maromizaha forest in Madagascar, a medium altitude evergreen rainforest [13] (Methods; Fig. 1A). On average, 72% of the individuals in each social group were sampled. Each sampled individual had previously been identified using natural marks, that is individual-specific traits that can be identified from a distance, such as the color and pattern of their fur patches, and their size. Sex identification was based on their singing patterns (assigned NA when sex identification was not possible due to early age). In addition, information was collected on individual age, reproductive pairs and respective progeny, and location data of the social groups (Methods; Table S1). As they frequently practice geophagy and this behavior has been hypothesized to contribute to acquisition of microbiome members that could facilitate digestion of plant material breakdown [20, 21], samples were also collected from geophagic sites, defined as where soil-eating events occurred (*N* = 7), together with non-geophagic sites nearby (*N* = 7) as control (Fig. 1A). Shotgun metagenomics was performed on all 32 samples (Methods; Table S1) with a median of 47 M reads/sample (IQR = [29.3, 70.0]) for the 29 samples with at least 1 M reads after quality control (three samples from geophagic soil were thus discarded, see Methods).

### Describing the *Indri indri* gut microbial tree of life

We performed metagenomic assembly and binning with a validated pipeline [48] (Methods) on the *I. indri* fecal samples we sequenced, together with those available in the literature (*N* = 4 samples in Greene *el al* [24]). We obtained a total of 342 medium and high-quality MAGs (256 of which from the present dataset), belonging to 48 different candidate species (species-level genome bins, SGBs [48]) (Methods). Of those, 9 were detected in the present dataset exclusively and 7 were exclusively assembled in Greene *et al.* [24], but the majority of species (66%, *N* = 32) were assembled in both datasets (Table S2) despite having only 18 samples from one population and 4 from another available. Therefore, despite the limited sample size, the unlikelihood of recent contact between the individuals in the two datasets—given that the Maromizaha Forest is approximately 25 km from the Ambatovy Conservation Zone in a straight line and separated by severe habitat fragmentation—suggests that the *I. indri* gut microbiome is mostly composed of a core set of species.

Only one of the species assembled, *Escherichia coli* (SGB10048), is well-characterized; in contrast, the other 47 had not previously been described (defined as uSGBs, species without cultivated representatives [48]). In addition, none of them belonged to genera with cultivated representatives: 14 of them had characterized representatives in the same family (6 species belonging to one same genus of *Coriobacteriaceae*, 5 to two genera of *Eggerthellaceae*, 2 to two genera of *Synergistaceae*, and 1 to a genus of *Synergistaceae*). Despite the uncertainty of assigning high-level taxonomies to very divergent species, we inferred that the remaining 34 uSGBs only had cultivated representatives at the phylum level (13 of *Bacteroidota*, 7 of *Firmicutes*, 5 of *Actinobacteria*, 3 of *Proteobacteria*, 2 of *Synergistetes*, 2 of Ca. *Melainabacteria*, and 1 of *Verrucomicrobia*) (Fig. 1B). This underscores that not only the species composing the *I. indri* microbiome remain virtually uncharacterized, but also the taxonomic lineages (genera, families) they belong to (Fig. 1).

### Core bacterial species make up the majority of the *Indri indri* microbiome

**Figure 2:**
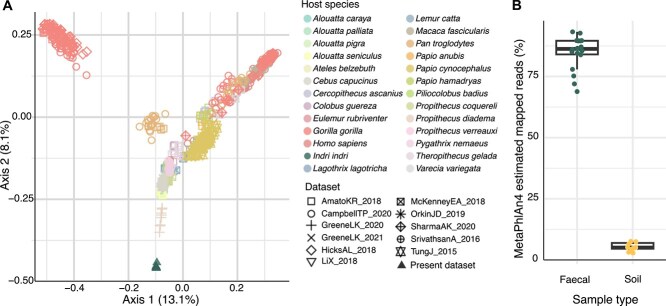
Profiling of *I. indri* and other NHP species gut microbiomes, and improvement of the *indri* microbiome mappability. (A) Profiling of *I. indri* and other primate fecal samples. PCoA on species-level Jaccard distances. Samples are colored by host species, and their shapes correspond to the dataset in which the samples were sequenced. (B) High mappability of the *I. indri* gut microbiome. After incorporating the assembled MAGs into a database for reference-based profiling, a 85% average mappability was achieved for *I. indri* fecal samples, whereas that of soil remained at 5%.

We used the catalog of MAGs obtained to expand the ChocoPhlAn [46] reference database and accurately profile *I. indri* microbiomes through reference-based profiling with MetaPhlAn. The database already contained MAGs from six metagenomic datasets of NHPs [31, 34–38] that were previously assembled [4], and in an attempt to obtain more complete profiling through microbial taxa that could be present in closely related host species, we expanded it with all other metagenomic datasets of NHPs (including other lemurs, sifakas, chimpanzees, and gorillas) that are currently available [3, 22, 24, 39–42] (*N* = 7 datasets; 5561 MAGs; Fig. 2A, Methods).

MetaPhlAn 4 profiling [46] on the expanded database resulted in an estimated average 85% (±6.94) of sequencing reads being assigned to SGBs (Fig. 2B), thus achieving a mappability that is comparable to that typically observed in human gut metagenomes [46, 48]. The relative abundance of species with cultivated representatives, that is kSGBs (Methods), was below 1%, corresponding to *E. coli* detected in 3 samples and *Cutibacterium acnes* (potential environmental or human contaminant) in 2 other ones. Four out of the five samples with these species belong to individuals in the three social groups (4MZ, 6MZ, and 8MZ) that reside either fully or partially within the Maromizaha ecotourism zone, with potential contact with tourists. Therefore, although *E. coli* has been detected in multiple host species [74, 75], acquisition of these two taxa from humans cannot be ruled out. The candidate species detected in *I. indri* gut microbiomes other than *E. coli* and *C. acnes* were extremely rarely found in other primates: out of the 476 samples profiled from 23 primate species, only two samples of red-shanked doucs (*Pygathrix nemaeus*) [39] harbored SGB98407 (phylum *Actinobacteria*) and SGB99124 (phylum *Proteobacteria*). In contrast, other primate species including humans had more in common (Fig. 2A).

The high host species specificity of the *I. indri* microbiome is complemented by a large core microbiome: consistent with the results obtained by metagenomic assembly in the previous section, most *I. indri*-specific bacterial species were consistently present in their microbiomes, revealing an unusually large common core set of species. Of the 28 species that were detected with this approach, 17 (60%) were found in all 18 individuals, 4 (14%) in all individuals but one, and all other species except for *E. coli* and *C. acnes* were detected in at least 66% of individuals sampled (N ≥ 12 individuals). We next inferred traits of the candidate species associated with the utilization of various substrates as carbon and energy sources, as well as oxygen requirements, cellular morphology, antibiotic susceptibility, proteolytic capabilities, and enzymatic activities [53] (Table S4, Methods). With the exception of *E. coli* (facultatively anaerobic), all candidate species were predicted to be strictly anaerobic. None were predicted to form spores, and 67% were classified as Gram-negative. Consistent with the predominantly plant-based diet of the *I. indri*, proteolytic activity was limited, while carbohydrate metabolism showed greater diversity (although metabolic variability was also observed).

Even though no eukaryotic species were detected by MetaPhlAn, we next assessed the presence of *Blastocystis* following the reports of its occurrence in NHP metagenomes [61] using a previously validated computational workflow (Methods). So far, *Blastocystis* has been detected in NHPs almost-exclusively when kept in captivity [61], suggesting potential transmission from humans [76]. Indeed, *Blastocystis* was not detected in any of the samples in this cohort of a limited sample size, although reference genomes are only available for the eight subtypes that are typically found in humans, and *I. indri* could thus host other subtypes that remain difficult to detect.

### Social group living is the main driver of the *Indri indri* microbiome

**Figure 3:**
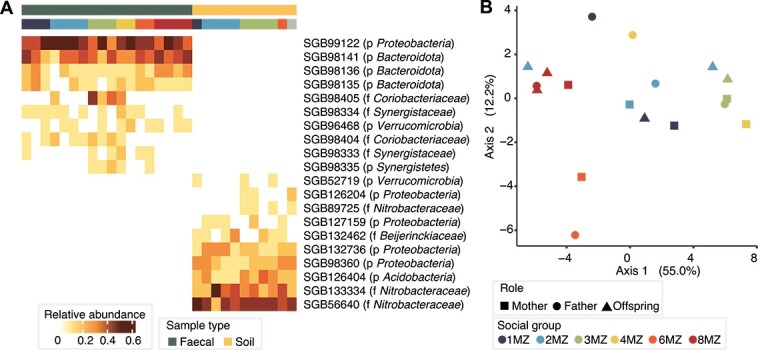
Bacterial species composition of *I. indri* fecal and soil samples. (A) The 10 most abundant candidate species in *I. indri* and in soil samples. The majority of the species (>99%) remain uncultivated (taxonomic label of the clade containing cultivated representatives in parentheses). No species co-occurred between fecal and geophagic soil sites. p: Phylum, f: Family. (B) Ordination of *I. indri* fecal samples (principal coordinates analysis on Aitchison distance of species-level microbiome composition). Social group emerged as the main covariate of species-level gut microbiome composition (*R*^2^ = 0.55, *P*_adj_ = 8.0e-05), whereas no other variables including age, sex, or social group role (mother, father, offspring) displayed significant associations.

Although the set of bacterial species detected was highly consistent across individuals, variations in relative abundances were detected (Fig. 3A). Social group emerged as the main covariate of the *I. indri* microbiome species-level composition (PERMANOVA on Aitchison distance, adj. *R*^2^ = 0.55, *P*_adj_ = 8.0e-05) (Fig. 3B), similar to what has been found in other primate species [31]. No other variables (age, sex, social group role, sequencing depth) were found significantly associated with beta diversity (*P*_adj_ > .05), but females displayed significantly higher alpha diversity than males (Simpson’s diversity index, *r* = 0.65, *P*-value = 7e-03). Although this pattern is also observed in humans and may result from hormonal effects, dietary and immune system differences, and higher gastrointestinal transit times [77], the preferential access to food sources of female *I. indri* (leading to feeding in higher branches of trees and richer grounds) in a female dominant society [78] could also contribute to their higher diversity. Age was not found significantly associated with alpha diversity, but the one infant included (0.5y, sex identification not possible due to early age) was the one displaying the lowest number of species (*N* = 20 vs average richness of other individuals *N* = 25.2), consistent with ongoing microbiome maturation in early ages.

At the functional level (profiling with HUMAnN, Methods), similar patterns were observed, with social group being the only variable displaying a significant association (PERMANOVA on Aitchison distance, *R*^2^ = 0.23, *P*_adj_ = 1.6e-02). Even though sex and age were not associated with functional alpha diversity, the youngest individual (who was likely still breastfed) also displayed the lowest number of functional pathways (*N* = 55 MetaCyc pathways vs average richness of other individuals *N* = 165.9). The most abundant pathways were related to amino acid [e.g. L-valine (VALSYN-PWY), L-isoleucine (ILEUSYN-PWY)], and nucleotide [e.g. inosine-5-phosphate (PWY-6123)] biosynthesis. Indeed, although seasonal variations are notable, the folivorous diet of *I. indri* might well be deficient in branched-chain amino acids, and microbial synthesis could compensate for this [24]. Pathways related to the degradation of leaves were also highly prevalent (>70% of individuals, none detected in the breastfed individual). These pathways included degradation of rhamnose (RHAMCAT-PWY), galactose (PWY-6317), galacturonate (GALACTUROCAT-PWY), and starch (PWY-6731). Rhamnose and galactose are constituents of hemicellulose, the second most abundant polysaccharide group in plants after cellulose, while D-galacturonate is the main monomeric constituent of pectin, and starch is the most abundant storage carbohydrate in plants [79]. Of note, hemicellulose, pectin, and starch are not only abundant in leaves but are also prevalent in various other plant tissues such as buds, bark, seeds, roots, flowers, and fruits (specially available during the wet season, when the samples in the present dataset were collected), so these pathways could also be relevant to digest the broader spectrum of plant food sources in the *I. indri* diet.

### No evidence of microbiome acquisition through geophagy

Soil eating behaviors are commonly observed in *I. indri*. These have been hypothesized to help neutralize toxic plant secondary compounds present in leaves, such as tannins, alkaloids, and polyphenols [13, 19]. Soil consumption could also lower gastric pH, act as antidiarrheal agent, supplement nutrient-poor diets, and counteract endoparasitic infections [20]. Another hypothesis is that geophagic soil contributes to populating the *I. indri* microbiome, potentially improving its ability to degrade leaf compounds, as shared fungal taxa were observed between fecal and geophagic soil samples [21]. Understanding whether soil consumption plays a role in shaping the *I. indri* microbiome could inform conservation efforts, potentially helping elucidate how they could successfully be kept under captivity when necessary. Accordingly, soil samples were included from the sites where social groups were observed to consume soil (*geophagic soil, N* = 7; *N* = 4 after excluding samples with less than 1 M reads after quality control; Fig. 1A), all located at the base of uprooted trees, exposing lower soil horizons; and from control sites (*non-geophagic soil*, *N* = 7) of similar characteristics and located less than 20 m away from each geophagic soil site.

Although soil samples were also assembled into MAGs and included in the expanded database, only 5.4% (±1.66) average mappability was achieved - markedly lower than that of the *I. indri* fecal samples (Fig. 2B). The fact that with reference-based profiling soil samples still displayed significantly higher microbial richness as compared to *I. indri* gut microbiomes (Wilcoxon rank-sum test, *r* = 0.51, *P*-value = 6.45e-3) attests to its high complexity together with the extent to which the soil microbiome remains underexplored [80], and the limitations of metagenomic assembly in such high-diverse sample types. Although the reduced number of samples certainly challenges power of detection, no significant differences in microbial diversity nor in species relative abundances was detected (Wilcoxon rank-sum tests, *P* > .05), not supporting that the *I. indri* select geophagic soil sites based on their microbial composition. What is more, only one of the 60 species found in soil samples was also detected in *I. indri* microbiomes (SGB98141, belonging to the phylum *Bacteroidota*; Table S2). This species was detected in all 18 *I. indri* samples but only in one soil sample, corresponding to a non-geophagic site. Even though mappability of the soil microbiome remains low, we expect that *Bacteria* conforming the now well metagenomically characterized *I. indri* microbiome would still be detected if they were present in soil at comparable relative abundances to other species detected. The lack of species co-occurrence between *I. indri* gut metagenomes and geophagic soil samples (and consequently no shared strains) does not support bacterial microbiome acquisition through geophagy.

The *I. indri* gut microbiome could exert strict control on the colonization by microorganisms acquired from diet. Indeed, we detected the presence of 11 different antimicrobial peptides (AMPs) in the assembled genomes (including mini-RNase 3, viscotoxin-A3, cypemycin, microcin B17, cytolysin, and multiple lysozymes; Methods), and found that 32% of the MAGs harbored at least one AMP (Table S5). All *I. indri* samples had at least one MAG encoding an AMP (median = 5, maximum = 11 AMPs/metagenomic sample), so exclusion mechanisms by the microbiome could well be in place. In addition, we assessed the distribution of the metabolic pathway leading to production of the secondary bile acids DCA and LCA, the main bile acids produced by bacteria that display antimicrobial activity (by disruption of bacterial membranes, inhibition of bacterial enzymes, or inhibiting colonization) [54, 55]. Although the full seven-step pathway of bile acid 7α-dehydroxylation (MetaCyc pathway PWY-7754, for conversion of the primary bile acids cholate or chenodeoxycholate to LCA or DCA, Methods*)* was not detected in any of the assembled genomes (which could be due to low sequence identity between the newly-assembled species and those in which the pathways have been characterized), a good portion (30%, 104 MAGs) encoded for at least 1 step of the pathway (Table S6). Still, soil-eating behaviors could complement coprophagy in facilitating the dispersion and acquisition of gut microorganisms from social partners (e.g. through spores), but the lower relative abundances of these microorganisms compared to other members of the soil microbiome may hinder their detection using metagenomic approaches.

### Extensive social microbiome transmission recapitulating host geography and interaction patterns

**Figure 4:**
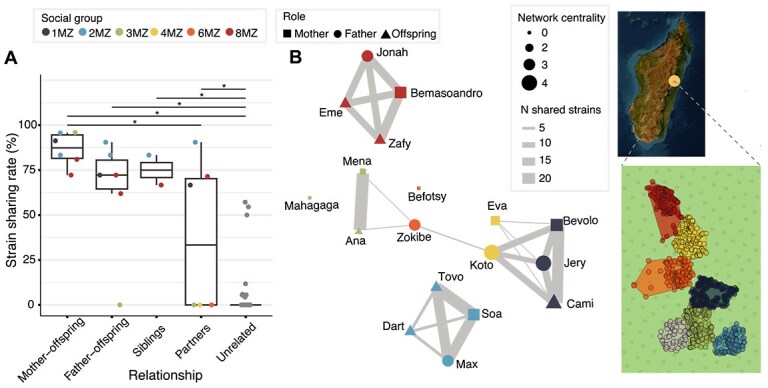
Microbiome strain sharing. (A) Individuals in the same social group display higher strain sharing rates than those in different social groups, regardless of their relationship. Kruskal-Wallis test, Chi2 = 85.3, *P*-value = 1.31e-17 with post-hoc Dunn tests, * corresponds to *P*_adj_ < .05 (Table S3). (B) Strain sharing patterns recapitulate social group membership and geography. Left: Unsupervised network on the number of shared strains (edges) between individuals (nodes). Edge width is proportional to the number of shared strains among individuals, while node size corresponds to the centrality (number of individuals each individual shares strains with). Unconnected nodes (individuals who do not share any strains with other individuals) are placed arbitrarily. Social group is identified by color and social group role by shape. Right: territory occupancy (annual MCP100) of the *I. indri* social groups 1MZ, 2MZ, 3MZ, 4MZ, 6MZ, 8MZ, and 9MZ (not sampled) during the year of 2018 in the Maromizaha forest of Madagascar.

We inferred microbiome transmission patterns among the individuals in the study through strain-level metagenomic profiling (Methods). Out of the 28 candidate species detected with MetaPhlAn using the custom database, 27 were successfully profiled at the strain level using StrainPhlAn. The highest interindividual strain sharing rates were detected between mothers and their offspring (median of 87%, 19 shared strains), followed by siblings (75%, 8.5 shared strains), fathers and their offspring (72%, 13 shared strains), and partners (33%, 6 shared strains) (Fig. 4A). Indeed, mothers are the primary caregivers, with infants clinging to their belly until they are four to five months old, when they move onto their back [81]. Although they start to demonstrate independence at around eight months, they are not fully independent until at least the age of two, so after microbiome seeding during delivery, close proximity is likely to promote mother to infant microbiome transmission as reported in humans [82, 83]. In addition, infants have been described to engage in coprophagy (fecal sample consumption) from maternal samples [84], which could further exacerbate mother-to-infant transmission. In contrast, at around the 20th week of life, geophagy progressively displaces coprophagy, which could promote acquisition of fecal matter from multiple social group members. The lower strain sharing among partners is due to the fact that three reproductive pairs exhibit close to 75% strain sharing rates (1MZ, 2MZ, 8MZ), whereas three other pairs (3MZ, 4MZ, 6MZ) share no strains (Fig. 4A). The absence of strain sharing among partners in group 3MZ can be attributed to a relatively recent takeover of the reproductive male of the group: following the loss of the previous reproductive male (Ratsy, not sampled) in 2015—likely from injuries sustained during a physical fight with a male from a neighboring group—Mahagaga (H_3MZ) assumed his role as an immigrant male [32]. Consequently, Mena (I_3MZ) had only been together with Mahagaga for 2 to 3 years, a much shorter time than her previous partnership. Indeed, Mahagaga did not share any strain with his 1 year old offspring (Ana, G_3MZ) either. In social group 6MZ, although not as clear, the reproductive male (Zokibe, S_6MZ) was found not to be the biological father of the eldest offspring (Tsiky, not included in our sampling) [15]. This suggests the possibility of either a previous reproductive male before Zokibe or an extra-pair copulation (EPC) event, which could potentially account for the absence of shared strains between the reproductive pair (Zokibe and Befotsy). In more recent years, Zokibe disappeared, with an immigrant male (from social group 4MZ) taking his place as the reproductive male of Befotsy (K_6MZ) in 2023. In contrast, a previous study that assessed genetic relatedness of 12 out of the 18 individuals in the study confirmed the monogamy, and discarded extra pair paternity of all other offspring [15].

Unlike group members (median of 72% strain sharing rate and 13 shared strains), unrelated individuals from different groups showed minimal strain sharing (median of 0 and 0%, respectively; averages of 0.3 and 1.5% respectively) (Fig. 4A), consistent with the minimal contact across social groups. The social structure of the *I. indri* microbiome was evident in a network representation on the strains shared (edges) across individuals (nodes) (Fig. 4B). The more isolated groups in the strain sharing network (2MZ and 8MZ) are also geographically located at the extremes, with fewer neighboring groups, whereas the remaining groups (with instances of shared strains across groups) live in closer territories. Indeed, the number of shared strains among individuals was negatively correlated with their geographical distance (based on the centroids of the social group territories, Methods) (Spearman’s test, rho = −0.46, *P*-value = 1.81e-09). Beside geographical distance, no other variables (sex, age) were found associated with network centrality measures (degree, eigenvector, and betweenness centrality, Methods), although females tended to display higher eigenvector centrality in the network than males (0.1 vs 0, 45 vs 36.5 median shared strains; Wilcoxon rank-sum test, *P*-value = 0.60), contrary to what has been reported in humans [85].

**Figure 5:**
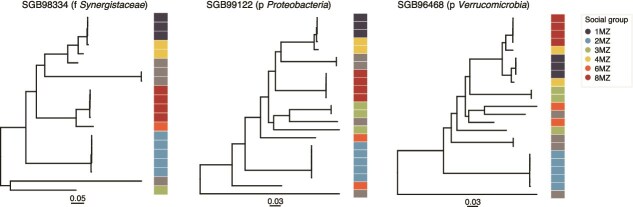
Phylogenetic trees of the candidate species displaying the highest social transmissibility. The first taxonomic label with cultivated representatives is noted (f: family, p: phylum). Nodes in gray correspond to *I. indri* fecal samples in the Greene *et al.* [24] dataset, for which information on social group membership was not available. Tree scale is noted under each tree. The trees of the other candidate species profiled at the strain level are shown in Figure S1.

We assessed whether the strains of certain candidate species are more frequently transmitted than others (species transmissibility, Methods). Median species transmissibility was 62.5% (meaning that in 62.5% of instances, when two members of a social group had a species in common, they shared the same strain), further indicating high microbiome transmission within social groups. Still, transmissibility ranged from 12.5% (SGB99124, phylum *Proteobacteria*) to 93.8% (SGB98334, family *Synergistaceae*) (Fig. 5). After inferring phenotypic traits of the candidate species (Table S4, Methods), no significant association between species transmissibility and Gram staining was found (Wilcoxon rank-sum test, *P-*value = .78), in contrast with larger studies in humans [25, 86]. Still, we found that average abundance of the candidate species were strongly and positively correlated with their transmissibility (Spearman’s test, rho = 0.75, *P* = 3.45e-05), favoring a mass-action model of transmission, a phenomenon that was not detected in previous studies [25, 87].

## Conclusion

In this study we tackle the so-far unexplored majority of the *I. indri* gut microbial diversity. We found the fecal microbiome of these NHPs to remain almost completely uncharted, with cultivated representatives available for fewer than 1% of its detected and inferred microbial species. Using metagenomic assembly on 18 newly sequenced samples together with 4 previously available [22], we reconstructed 342 medium- and high-quality MAGs, representing 48 different candidate species. Among these, only *E. coli* is well-characterized, while the other species were mostly specific to *I. indri* and not detected in other publicly available samples of primate microbiomes. The majority of these species did not have cultivated representatives in the same genus or family, underscoring the extent to which clades of the microbial tree of life remain virtually unexplored. After integrating the newly identified genomes into a custom reference database, we achieved an average of 85% estimated mappability, showcasing the power of metagenomics approaches to survey the so-far uncharacterized fraction of wild animal microbiomes.

The *I. indri* microbiome seems to largely consist of a common core set of bacterial species (all newly identified species were detected in >66% of individuals in the two populations included in this study). This is in contrast with other primate species microbiomes, which display much higher variation in species occurrence [3]. Our study is certainly limited by the number of samples, and while the sample size is well aligned with previous studies using shotgun metagenomic sequencing on wild NHPs (6 samples of *P. femoralis* [39]*,* 18 samples of *V. variegata, L. catta,* and *P. coquereli* [40], 12 samples of *P. diadema* and *I. indri* [24], 23 samples of *G. gorilla* [42], 18 samples of *P. troglodytes* and 28 of *G. gorilla* [41], and 11 samples of *P. coquereli* [22]), future works should focus on providing larger-scale datasets together with longitudinal sampling to understand the temporal variation of the *I. indri* microbiome. Still, the fact that most species were detected in two independent populations suggests that the main members of the *I. indri* microbiome were successfully profiled. In line with studies in other primates [26–28, 31], social group belonging emerged as the main determinant of this lemur’s microbiome composition, both at the taxonomic and functional levels. We found evidence for extensive interindividual transmission within social groups, positioning social physical interaction among group members (such as grooming, scratching, or playing) as a main driver of microbiome acquisition and, consequently, composition. Species in *I. indri* microbiomes were predominantly predicted to be anaerobic and non-spore formers, phenotypic traits that were previously found to be characteristic of socially structured taxa [31]. In line with the rare intergroup encounters and low territory overlap, strain sharing between unrelated individuals was minimal and negatively correlated with geographical distance. Indeed, the *I. indri* social structure, characterized by monogamy and pair living, is unlikely to promote microbial strain exchange across groups. Additionally, increasing habitat fragmentation is expected to further constrain the acquisition of microbial diversity beyond the host’s social environment. We did not find evidence for microbiome acquisition through geophagy, as neither strains nor species co-occurred in geophagic soil and fecal samples, although the number of soil samples was suboptimal. Soil-eating behaviors, in addition to aiding the digestion of plant polysaccharides and facilitating micronutrient acquisition, may still promote the acquisition of gut microbiome members. Indeed, coprophagy from mother’s feces is progressively replaced by geophagy in infants, which could contribute to microbiome maturation by acquisition of microbiome members from other social partners. Ingested soil could contain traces of fecal matter with microbial metabolites, spores, or the microorganisms themselves at low levels, preventing their detection in metagenomic analyses. This would contribute to the high social transmission of the microbiome observed.

Overall, while larger studies are needed to confirm our findings, they underscore the crucial role of the social landscape in shaping the *I. indri* microbiome, as evidenced by its proportionally very large core microbiome and high rates of transmission within social groups. The present provides a foundation for future research to understand the impact of disease, habitat degradation, anthropogenic disturbances, and captivity on the gut microbiome of the critically endangered *I. indri*, highlighting the importance of considering the social microbiome in efforts to mitigate the species risk of extinction.

## Supplementary Material

Figure_S1_wraf136

Indris_Supplementary_Tables_wraf136

## Data Availability

Shotgun metagenomics sequencing data are available at the European Nucleotide Archive under accession number (PRJEB89425). Metadata are available in Table S2.

## Resource availability

### Materials availability

This study did not generate new unique reagents.

### Code availability

All the software used in this study are available in the MetaPhlAn4 package (which includes StrainPhlAn4 and the script for strain transmission inference), available at http://segatalab.cibio.unitn.it/tools/metaphlan with the open-source code at https://github.com/biobakery/MetaPhlAn.
