# Supplementary material for: Bacterial transmission within social groups shapes the underexplored gut microbiome in the lemur Indri indri
Source: ISME J. 2025 Jul 25;19(1):wraf136. doi: 10.1093/ismejo/wraf136 (PMC12596700; doi:10.1093/ismejo/wraf136)
Supplement: Figure_S1_wraf136 [file figure_s1_wraf136.pdf]

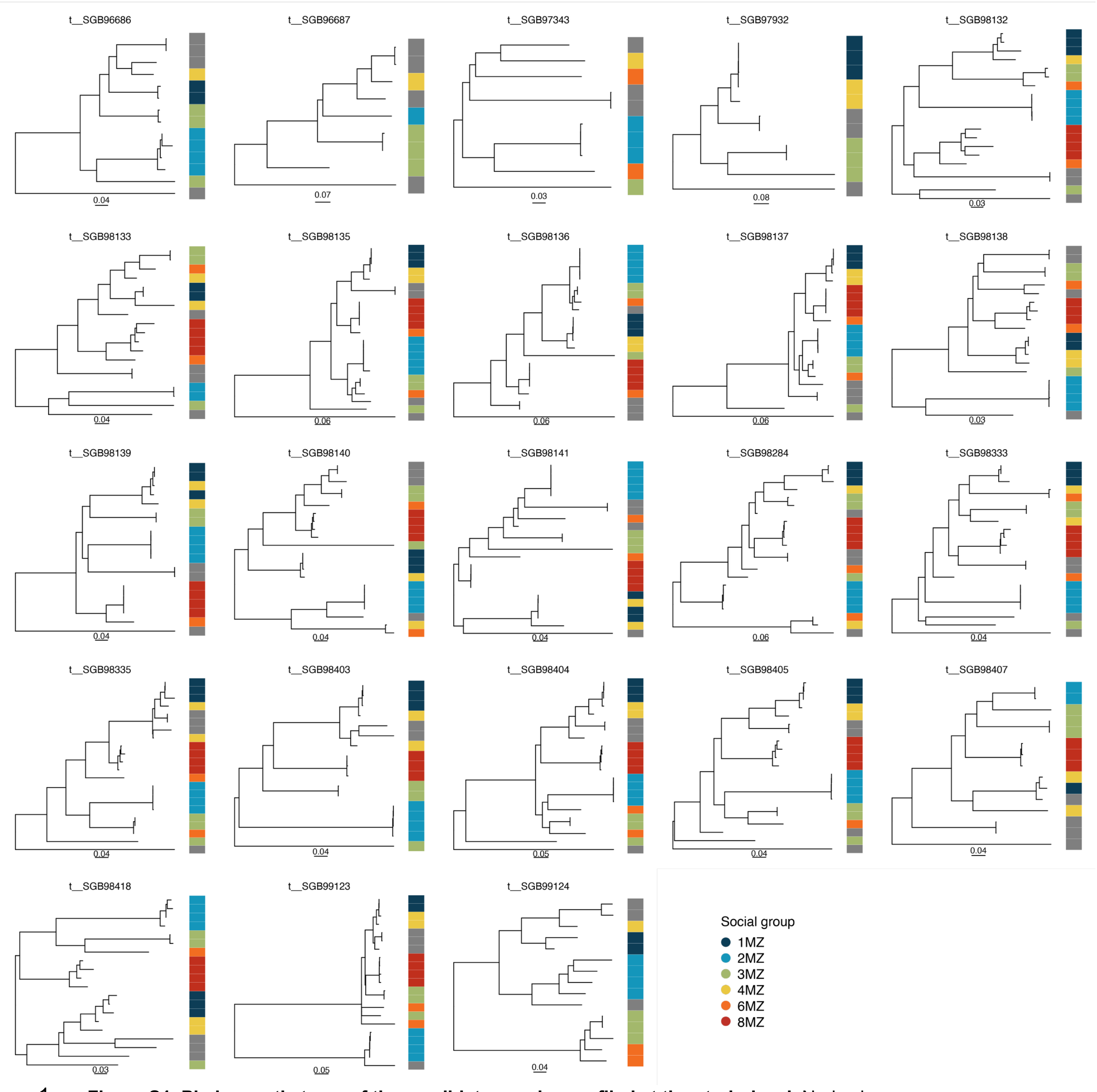

Figure S1. Phylogenetic trees of the candidate species profiled at the strain-level. Nodes in grey correspond to *Indri indri* faecal samples in the Greene *et al[24]* dataset, for which information on social group membership was not available.
